# Supplementary material for: MicroRNA-223 Suppresses Human Hepatic Stellate Cell Activation Partly via Regulating the Actin Cytoskeleton and Alleviates Fibrosis in Organoid Models of Liver Injury
Source: Int J Mol Sci. 2022 Aug 19;23(16):9380. doi: 10.3390/ijms23169380 (PMC9409493; doi:10.3390/ijms23169380)
Supplement: Supplementary file 1 [file ijms-23-09380-s001.zip › ijms-1831823-supplementary.pdf]

Supplemental information

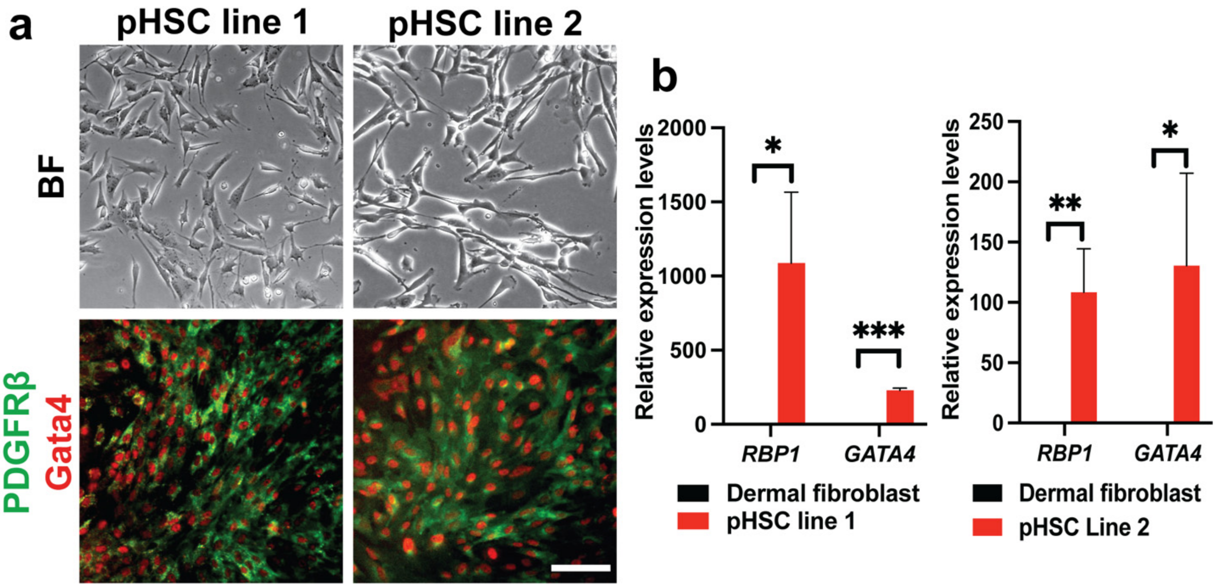

Figure S1. Characterization of primary human HSC (pHSC) lines

(a) Brightfield (BF) images show cell morphology of HSCs from two independent donors, and immunofluorescence images show staining of a generic mesenchymal cell marker, PDGFRβ, and an HSC-specific transcription factor, Gata4. Scale bar: 100 μm.

(b) qRT-PCR analysis of HSC-specific markers in HSCs compared with dermal fibroblasts. Data presented as mean ± SD and expressed relative to those of dermal fibroblasts (set as 1.0). N = 3 replicates per group from two independent experiments. Student's t-test; *=p<0.05, **=p<0.01 and ***=p<0.001. RBP: Retinol-binding protein.

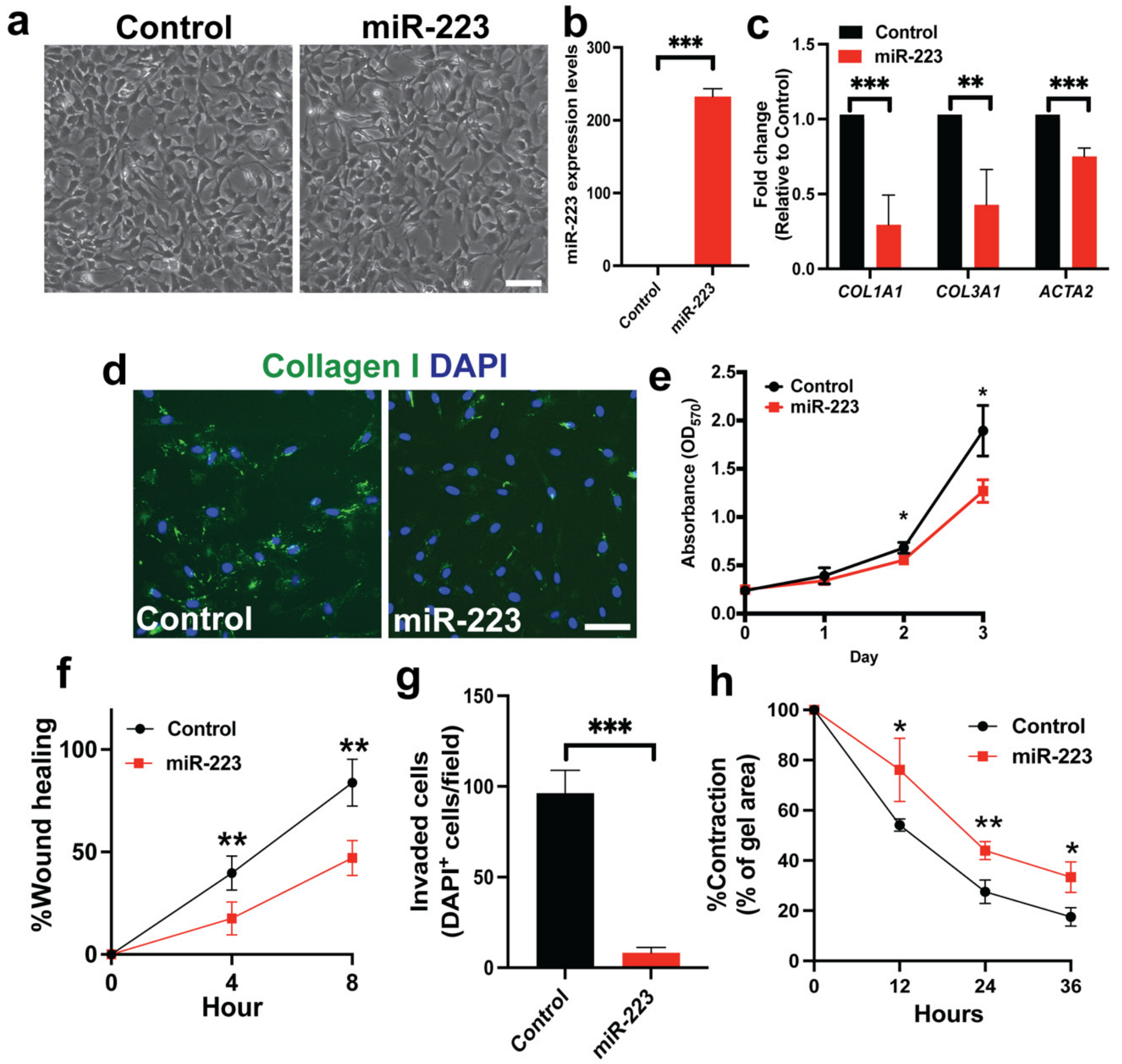

Figure S2. MicroRNA-223 suppresses activation phenotypes in an independent line of primary human HSCs.
(a) Cell morphology of control and miR-223 HSCs
(b) Lentivirus-mediated overexpression of miR-223 in pHSCs measured by qRT-PCR.
(c) qRT-PCR analysis of fibrotic gene transcripts upon overexpression of miR-223.
(d) Expression of type I collagen in control and miR-223 pHSCs by immunofluorescence.
(e) Cellular proliferation of control and miR-223 HSCs as determined by the MTT assay.
(f) Wound healing assay of control and miR-223 HSCs.
(g) Quantification of invaded cells after seeding in a Matrigel-coated transwell for 24 hours with chemoattractant (2% FBS). Invaded cells were counted from 10 random microscope fields for each group.
(h) Collagen matrix contraction measured at 12-hour intervals after the lattices were dislodged.
Data presented as mean ± SD and expressed relative to those of control HSCs (set as 1.0) for qRT-PCR experiments. N = 3-4 replicates from at least two independent experiments. Student's t-test; *=p<0.05, **=p<0.01 and ***=p<0.001. Scale bars, 100 μm. DAPI: 4′,6-diamidino-2-phenylindole.

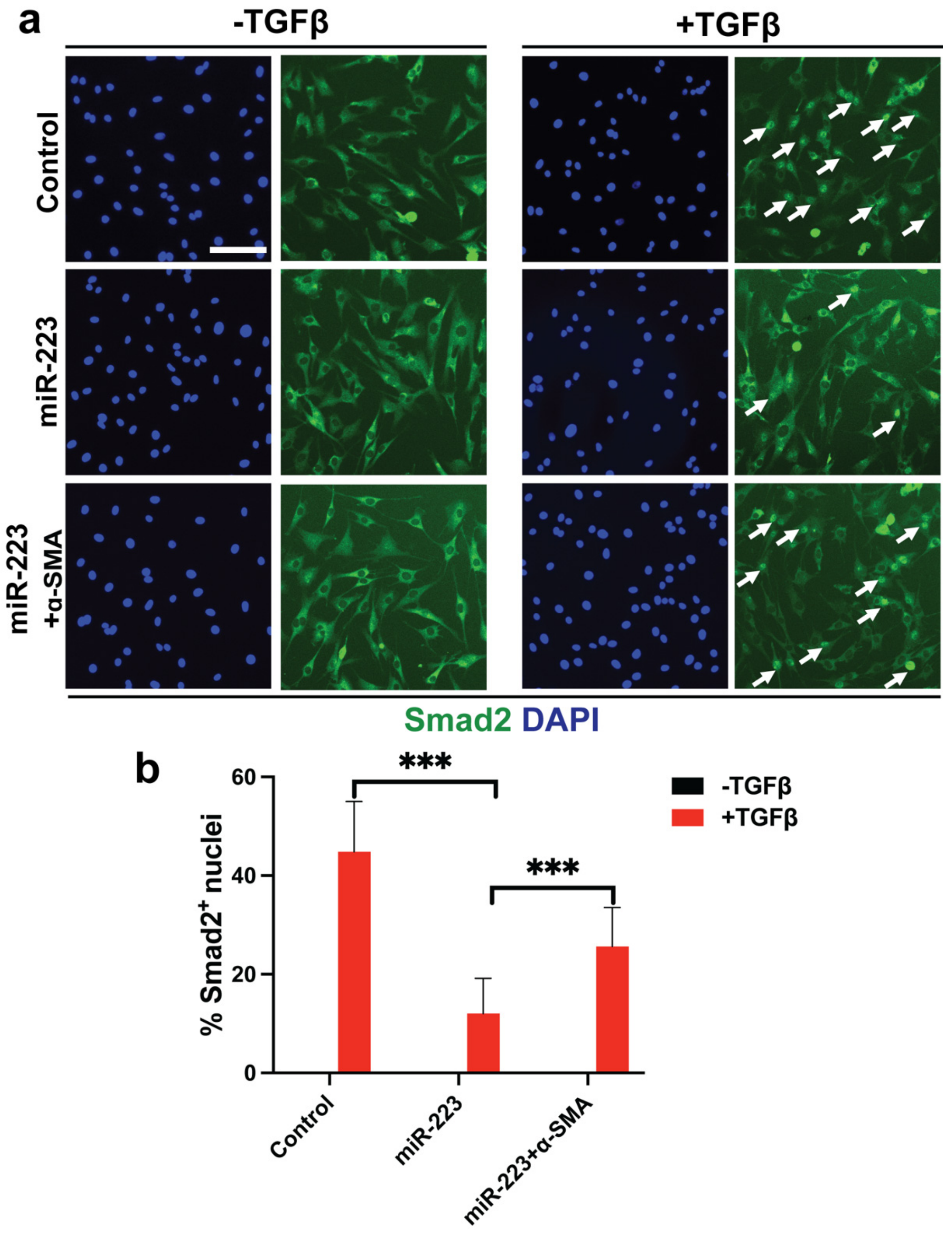

Figure S3. Overexpression of α-SMA in miR-223 HSCs partially restores nuclear translocation of Smad2.

(a) Immunofluorescence of Smad2 for indicated HSCs before and after TGFβ stimulation. White arrows indicate cells with nuclear translocation of Smad2. Scale bars, 100 μm. DAPI: 4′,6-diamidino-2-phenylindole.

(b) Quantification of nuclear Smad2+ cells were counted from 10 random microscope fields for each group. Data presented as mean ± SD. Student's t-test; ***=p<0.001.

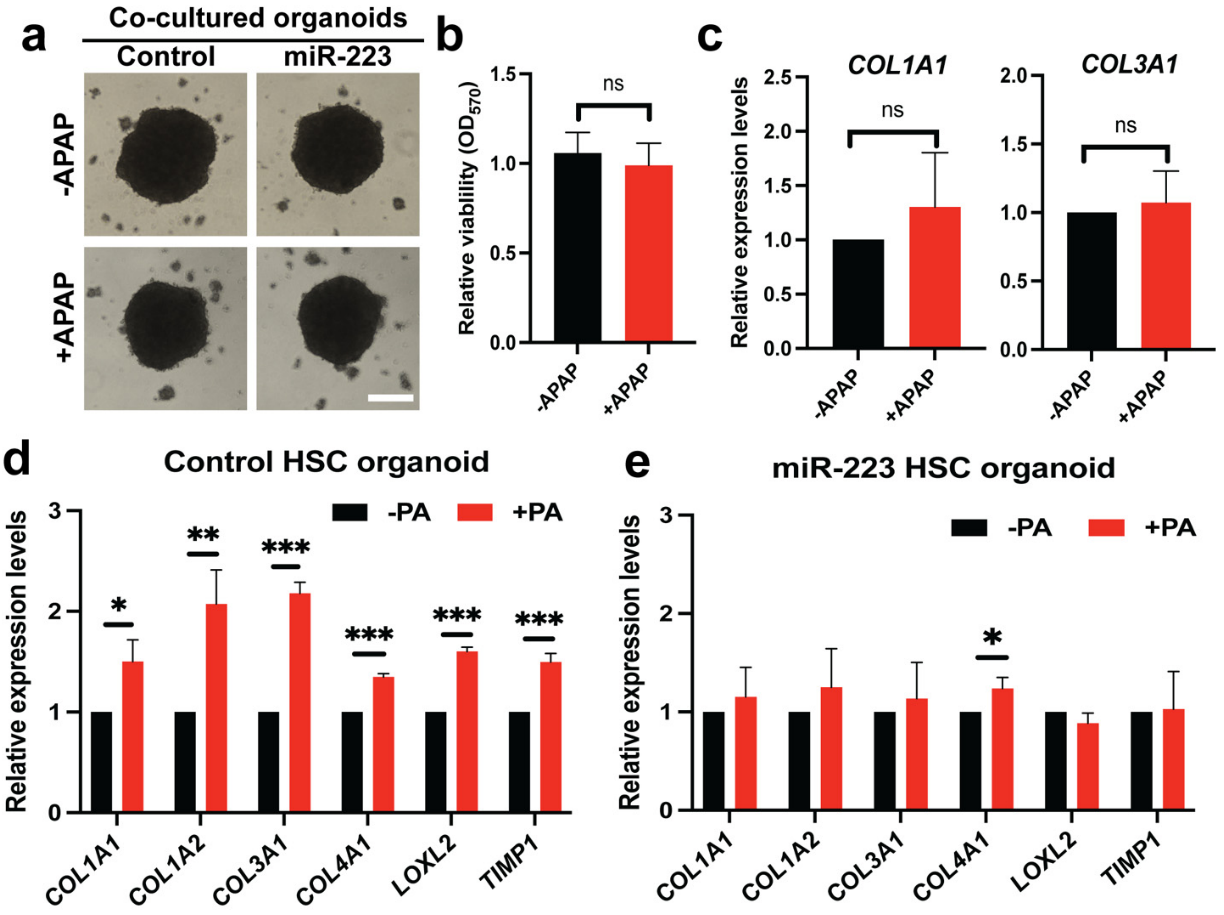

Figure S4. Supplemental experiments related to injury models of co-cultured hepatic organoids
(a) Morphology control and miR-223 organoids with or without APAP treatment. Scale bars, 100 μm.
(b) Cell viability of HSCs with or without APAP treatment determined by the MTT assay.
(c) qRT-PCR analysis of fibrotic gene transcripts from HSCs with or without APAP treatment.
(d, e) qRT-PCR analysis of fibrotic gene transcripts from control and miR-223 organoids with or without palmitic acid (PA) treatment.
Data presented as mean ± SD and expressed relative to those of untreated controls (set as 1.0) for qRT-PCR experiments. N = 3-4 replicates from at least two independent experiments. Student's t-test; *=p<0.05, **=p<0.01 and ***=p<0.001. APAP: N-acetyl-para-aminophenol. MTT: 3-(4,5-dimethylthiazol-2-yl)-2,5-diphenyl-2H-tetrazolium bromide.

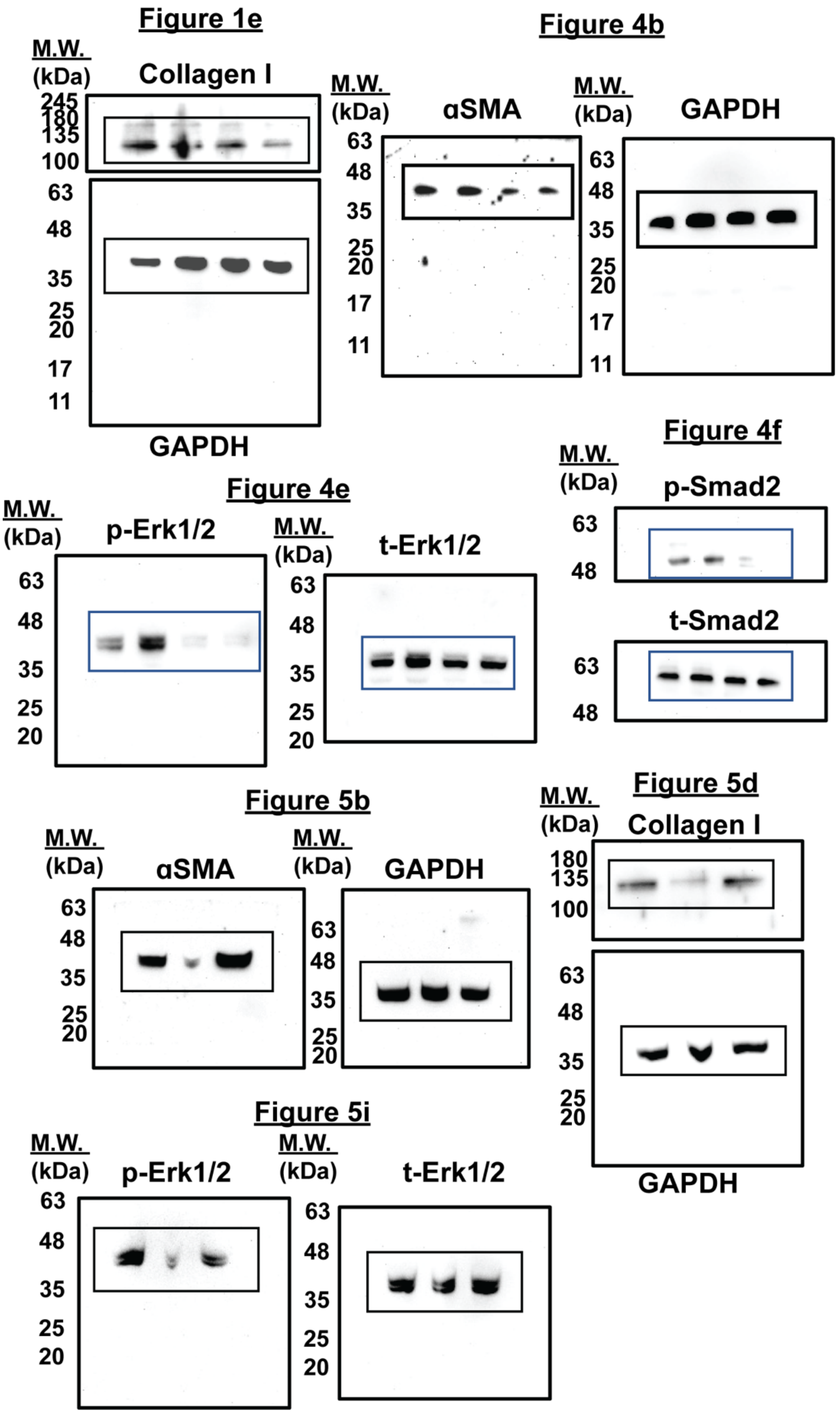

Figure S5. Uncropped immunoblots

Table S1: Primer sequences used in this study

Cloning primers:

Fwd_EcoRI_miR-223	gctgaattcccatagctacaggactcagg
Rev_BamHI_miR-223	cgaggatccaagagcacttccattcctgg
Fwd_EcoRI_ACTA2	gctgaattcgccgccaccatgtgtgaagaagaggacagc
Rev_BamHI_ACTA2	cgaggatccttagaagcatttgcggtgga
Top_WT_3'UTR_ACTA2	cgcggccgctgtgtttttaataaatctgaac
Bottom_WT_3'UTR_ACTA2	tcgagttcagatttattaaaaaacacagcggccgcgagct
Top_MUT_3'UTR_ACTA2	cgcggccgctgtgtttttaccccttacctac
Bottom_MUT_3'UTR_ACTA2	tcgagtaggtaaggggtaaaaacacagcggccgcgagct
Top_miR-223_pSilencer	gatccgtgtcagtttgtcaaataccccattcaagagatggggtatttg acaaactgacatttttggaaa
Bottom_miR-223_pSilencer	agcttttccaaaaaatgtcagtttgtcaaataccccatctcttgaatg gggtatttgacaaactgacacg

Underlined sequences indicate restriction sites used for cloning

qRT-PCR primers:

Fwd_U6	ctcgcttcggcagcaca
Fwd_miR-223	tgtcagtttgtcaaatacccca
Rev_universal_miR	gcagggtccgaggtattc
F_RPL19	gctctttcctttcgctgct
R_RPL19	cattggtctcattggggtct
F_COL1A1	cccaaggcttccaaggtc
R_COL1A1	ggacgaccaggttttccag
F_COL1A2	gagtccgaggacctaatgga
R_COL1A2	aggggaaccaggaagacct
F_COL3A1	tggtggtaaaggcgaaatg
R_COL3A1	agtccaggagcaccattagc
F_COL4A1	gggtgaaccaggaaaaattg
R_COL4A1	ggaaagcctcggtctcctt
F_ACTA2	ctgttccagccatccttcat
R_ACTA2	tcatgatgctgttgtaggtggt
F_LoxL2	ttggaggacacagaatgtgaa
R_LoxL2	agtcgatgtcatggcggta
F_TIMP1	ctgttgttgctgtggctgat
R_TIMP1	aacttggccctgatgacg
F_CCND1	cacttgcatgttcgtggcct
R_CCND1	gaggaagtgttcaatgaaatcgt
F_CCNE1	gggcgtcgctgatgaagatg
R_CCNE1	gcccgctgctctgcttctta
F_GFAP	acccagcaactccaactaac
R_GFAP	ttctctccttcctcctcattct
F_RELN	ttcctaacgcagcactaacc
R_RELN	gagacatgacgggccaatataa
F_TBX20	aaggaggcgacggagaac
R_TBX20	gcacaggacgacttctccac
F_RSPO3	gtcagaagggagaacgagga
R_RSPO3	tttgctgtcaggtattgcttct

F_NGFR	tcatccctgtctattgctcca
R_NGFR	tgttctgcttgcagctgttc
F_PCDH7	ttgagactggcttgttgctg
R_PCDH7	ccacccagacaaacgtgag
F_VEGFB	ctggccaccagaggaaagt
R_VEGFB	ccatgagctccacagtcaag
F_KIRREL	tgccaccatcatctggttc
R_KIRREL	ttcccatccttcagcaattc
F_HGF	gattggatcaggaccatgtga
R_HGF	ccattctcattttatgttgctca
F_TLN2	tgatttctgctgcccggatgg
R_TLN2	agcatttcctgccgcctgtag
F_LRAT	ctggaggtggtgtctttactac
R_LRAT	actgttcctccctttgtcttc
F_GATA4	ggaagcccaagaacctgaat
R_GATA4	gttgctggagttgctggaa
F_RBP1	aggcatagatgaccgcaagt
R_RBP1	ttctgcacacactggagctt
